# Bio-control of soil-borne virus infection by seed application of *Glycyrrhiza glabra* extract and the rhamnolipid Rhapynal

**DOI:** 10.1007/s00425-024-04529-5

**Published:** 2024-09-13

**Authors:** Viktoria Fomitcheva, Claudia J. Strauch, Sabine Bonse, Petra Bauer, Thomas Kühne, Annette Niehl

**Affiliations:** Institute for Epidemiology and Pathogen Diagnostics, Julius Kühn Institute (JKI), Federal Research Centre for Cultivated Plants, Messeweg 11-12, Brunswick, Germany

**Keywords:** Bio-substance, Environmentally friendly virus control, Plant protection, *Polymyxa betae*, *Polymyxa graminis*, Seed-treatment, Soil-treatment, Sugar beet, Wheat

## Abstract

**Main conclusion:**

Seed-application of the natural products protects sugar beet and wheat plants against infection with plasmodiophorid-transmitted viruses and thus may represent an efficient, environmentally friendly, easy and cost effective biocontrol strategy.

**Abstract:**

In times of intensive agriculture, resource shortening and climate change, alternative, more sustainable and eco-friendly plant protection strategies are required. Here, we tested the potential of the natural plant substances *Glycyrrhiza glabra* leaf extract (GE) and the rhamnolipid Rhapynal (Rha) applied to seeds to protect against infection of sugar beet and wheat with soil-borne plant viruses. The soil-borne *Polymyxa betae-* and *Polymyxa graminis*-transmitted viruses cause extensive crop losses in agriculture and efficient control strategies are missing. We show that GE and Rha both efficiently protect plants against infection with soil-borne viruses in sugar beet and wheat when applied to seeds. Moreover, the antiviral protection effect is independent of the cultivar used. No protection against *Polymyxa sp.* was observed after seed treatment with the bio-substances at our analysis time points*.* However, when we applied the bio-substances directly to soil a significant anti-*Polymyxa graminis* effect was obtained in roots of barley plants grown in the soil as well as in the treated soil. Despite germination can be affected by high concentrations of the substances, a range of antiviral protection conditions with no effect on germination were identified. Seed-treatment with the bio-substances did not negatively affect plant growth and development in virus-containing soil, but was rather beneficial for plant growth. We conclude that seed treatment with GE and Rha may represent an efficient, ecologically friendly, non-toxic, easy to apply and cost efficient biocontrol measure against soil-borne virus infection in plants.

**Supplementary Information:**

The online version contains supplementary material available at 10.1007/s00425-024-04529-5.

## Introduction

In the face of climate change, decreasing biodiversity, resource shortening and increased demand for human nutrition, a more sustainable agriculture is urgently needed. Natural plant protection substances emerge as promising candidates to promote plant growth and resilience while being natural, ecofriendly and biodegradable.

The extract of *Glycyrrhiza glabra* L. (GE) and the bio-surfactant Rhapynal (Rha) are bio-substances which have proven to efficiently protect plants against infection with microbial pathogens through foliar application (Schuster et al. 2010; Hermann et al. 2022; Thomidis and Prodromou 2022; Porsche et al. 2023). Whether these substances also efficiently act as plant protection agents through application to seeds and whether these substances can protect against plant virus infection remains unexplored. *Glycyrrhiza glabra* (liquorice) is a perennial plant belonging to the family Fabaceae. It is native to Asia and the Mediterranean region. Its root is known for long as therapeutic in traditional medicine. Also the leaf extract of *Glycyrrhiza glabra* has been shown to display antifungal and antibacterial properties (Schuster et al. 2010; Scherf et al. 2012; Hermann et al. 2022; Porsche et al. 2023). These antifungal and antibacterial properties have been associated with the direct cytotoxic activity of the contained polyphenolic compounds and stimulation of immunity.

Rhamnolipids (RLs) are amphiphilic lipopolysaccharides which are known for biocompatibility, biodegradability and low toxicity (Liu et al. 2018; Luzuriaga-Loaiza et al. 2018). RLs are produced by various bacterial species such as *Pseudomonas* spp. and *Burkholderia* spp. (Toribio et al. 2010; Crouzet et al. 2020; Soberón-Chávez et al. 2021; Dabaghi et al. 2023). They affect flexibility of microbial surfaces, bacterial motility, the development and disruption of biofilms, bacterial virulence and antimicrobial activity (Abdel-Mawgoud et al. 2010; Chrzanowski et al. 2012). RLs have also been identified as potential molecules for stimulating immunity in animal cells (Andrä et al. 2006; Bauer et al. 2006; Howe et al. 2006; Vatsa et al. 2010). In plants, RLs have been shown to exhibit antimicrobial activity and also to act as elicitors of defense and induce resistance to bacterial, fungal and nematode pathogens (Varnier et al. 2009; Vatsa et al. 2010; Sanchez et al. 2012; Goswami et al. 2015; Bredenbruch et al. 2023; Kossmann et al. 2023). The bio-substance GE can be cost-efficient and easily produced by ethanolic extraction from leaves and RLs can be produced in bioreactors by microorganisms (Tiso et al. 2017, 2020).

Against plant virus infection no direct control measures are possible. Plant virus infection is usually combatted by strict hygiene measures and growth of resistant plants (Jones 2006; Kühne 2009; McGrann et al. 2009). Moreover, for insect-transmitted viruses insecticides can help to limit virus infection by limiting the number of insect vectors. Viruses transmitted by soil-borne pathogens are specifically difficult to combat. *S*oil-borne viruses transmitted by plasmodiophorids of the genus *Polymyxa* can cause extensive damage to susceptible crop plants, reaching 50% depending on environmental parameters (Kanyuka et al. 2003; Kühne 2009; Ordon and Perovic 2013; Tamada et al. 2021; Yang et al. 2022). Examples for economically important viruses transmitted by *Polymyxa* species are the sugar beet-infecting beet necrotic yellow vein virus (BNYVV, Benyvirus) which may occur together with beet soil-borne virus (BSBV, Pomovirus) and beet virus Q (BVQ, Pomovirus), (Kastirr and Richert-Pöggeler 2018; Nouayti et al. 2019; Weiland et al. 2020), the cereal infecting soil-borne wheat mosaic virus (SBWMV, Furovirus) and the barley-infecting yellow mosaic bymoviruses barley yellow mosaic virus (Bymovirus) and barley mild mosaic virus (Bymovirus).

These viruses transmitted by *Polymyxa* species are contained in resting spores formed by the vector and can remain infectious in soil for many years. Thus, conventional methods to control these soil-borne diseases are inefficient. Resistant plants are a means to efficiently control infection. However, efficient resistance genes are scarce and for some resistance genes the resistance is easily overcome by the viruses (Kanyuka et al. 2004b; Bass et al. 2006; Maccaferri et al. 2011; Li et al. 2016; Decroës et al. 2022; Liebe et al. 2023; Okada et al. 2023). Hence, new, effective and environmentally friendly approaches to control the viruses are urgently needed.

Here, we explored whether GE and Rha applied to seeds protect sugar beet (*Beta vulgaris*) and wheat (*Triticum aestivum*) against infection with the soil-borne viruses BNYVV, BVQ, BSBV and SBWMV and their vectors, *Polymyxa betae* and *Polymyxa graminis*. We also investigated the influence of the bio-substances applied to seeds on plant germination, growth and development.

## Materials and methods

### Plant material

Sugar beet (*Beta vulgaris* L.) seeds of the resistant varieties Lisanna and Hannibal and of the susceptible genotype MS 133E15250 were kindly provided by KWS Saat SE & Co. KGaA (Einbeck, Germany) and Strube Research GmbH & Co. KG (Rommerskirchen, Germany), respectively. Sugar beet plants were grown from seeds in field soil under climate chamber conditions with 14 h light (10 kLux) at 22 °C and 10 h night at 18 °C. The winter wheat (*Triticum aestivum* L.) varieties Kamerad and Chevignon were obtained from Hauptsaaten (Cologne, Germany). The historical variety Alcedo was from JKI stocks, the variety Reflection was obtained from Syngenta (Frankfurt, Germany). Wheat plants were grown from seeds in field soil in the greenhouse with 16 h light (300 µmol m^2^ s^−1^) at 20 °C and 8 h dark at 15 °C. Barley (*Hordeum vulgare* L*.*) plants cv. Keeper were obtained from KWS. Barley plants were grown in the climate chamber from seeds in field soil with 8 h light at 12 °C.

### Seed treatments

GE stock solution containing an extract made from 40 g leaves in 100 mL was kindly provided by Trifolio GmbH (Lahnau, Germany). The biosurfactant Rhapynal was obtained by Biotensidon (Karlsruhe, Germany) as aqueous solution at a concentration of 200 g L^−1^. Pelleted sugar beet seeds were incubated for 15 min, 16 h or 8 h at room temperature in solutions containing undiluted GE or GE diluted 1:5 and 1:10 in triple-distilled water. For treatment with Rha, pelleted sugar beet seeds were incubated for 15 min, 16 h or 8 h at room temperature in aqueous solutions containing 200 g L^−1^, 100 g L^−1^, 40 g L^−1^, 20 g L^−1^, or 2 g L^−1^ Rha. Non-pelleted sugar beet seeds were incubated 16 h or 8 h, respectively, in GE solution diluted 1:10 in triple-distilled water and in an aqueous solution containing 40 g L^−1^ Rha. Winter wheat seeds were incubated for 9 h at room temperature in GE solutions diluted 1:10, 1.20, 1:50 or 1:100 in triple-distilled water. For treatment with Rha, winter wheat seeds were incubated for 9 h at room temperature in aqueous solutions containing 100 g L^−1^, 20 g L^−1^, 4 g L^−1^, or 2 g L^−1^Rha. Control seeds were incubated in triple distilled water.

### Virus-containing soils

Soil from Mintraching (Germany) contained *P. betae*, BNYVV (B-Pathotype), BVQ and BSBV. Soil from Pithiviers (France) contained *P. betae*, BNYVV (P-Pathotype), BVQ and BSBV. Soil from Elxleben (Germany), contained *P. graminis* and SBWMV and soil from Bornum (Germany), contained *P. graminis*, Japanese soil-borne wheat mosaic virus and barley mild mosaic virus.

### Seed germination and analysis of plant development

To investigate plant development, GE- and Rha-treated seeds or water-treated control seeds were placed into field soils and plants were grown from seeds under climate chamber conditions. Seedlings were taken out of the soil carefully at different times after sowing, roots were washed thoroughly under running water and photographs of the phenotypes were taken. For the quantification of root biomass, roots were dried at room temperature for two days after washing. Washed and dried roots were weighed six weeks after sowing into soil from Pithiviers. Roots of two to five plants per genotype were weighed together, and the average weight per plant calculated. Data for the three genotypes analyzed, Lisanna, Hannibal and MS133E15250 were averaged and standard deviation (SD) calculated.

### Soil-treatment with GE and Rha and detection of *P. graminis* DNA in barley roots by quantitative real-time PCR

Barley cv. Keeper seeds (20 per pot) were planted into soil from Bornum and grown during 14 weeks in a climate chamber. The soil of pots with 11 cm in diameter were irrigated once a week with 15 mL of water or GE diluted 1:100 in water, 20 g L^−1^ Rha or 2 g L^−1^ Rha, respectively. After 14 weeks, seedlings were carefully removed from the soil and roots were thoroughly washed under running water. DNA extraction was performed with the roots of the plants using the DNeasy plant mini kit (Qiagen, Hilden, Germany) according to the manufacturer’s instructions. qPCR was conducted with primers PxRealF, PxRealR and probe PxRealP (Ward et al. 2005) (Table S1). 10 µL reaction solution contained 0.6 µL PxRealF, 0.2 µM PxRealR and 0.15 µM PxRealP, and 5 µL 2xLuna Probe Kit (NEB, Ipswich, MA, USA). Reaction conditions in the qTower2.2 thermocycler (Analytik Jena AG, Germany) were 1 min at 95 °C and 40 cycles of 15 s at 95 °C and 30 s at 60 °C.

### RNA extraction from roots

For detection of sugar beet viruses and SBWMV, RNA from root tissue was isolated using Tri reagent (Bio&Sell, Feucht, Germany). Filamentous roots were washed under running water and frozen in liquid nitrogen. The root material was then ground with mortar and pestle to a fine powder and 100 mg incubated with 500 µL of Tri reagent. After 5 min exposure at room temperature the solution was clarified by centrifugation at 12.000 g for 2 min. The supernatant was transferred to RNase-free Eppendorf tubes and mixed with 100 μL 5 M NaCl and 300 μL chloroform. After centrifugation for 10 min at 12.000 g, 4 °C, the upper, aqueous phase was transferred to a new tube and incubated for 30 min with an equal volume of isopropanol. After centrifugation at 12.000 g for 10 min, 4 °C, the RNA pellet was washed twice with 70% ethanol and air-dried for 10 min. Finally, it was dissolved in 50 μL of RNase-free water.

### RNA extraction from soil

For the detection of *P. betae*, RNA from soil samples was isolated using FastRNA® Pro-Soil-Direct Kit (MP Biomedicals, Irvine, CA, USA) according to the manufacturer’s instructions. 0.5 g of well-mixed and crushed soil was used per sample. For lysis of all biological components the soil samples were transferred to tubes containing application-specific microspheres (beads) of different size. After adding of 1 mL RNA Soil Lysis Solution, the tube was replaced in a reactor vessel recommended for the rotor–stator homogenizer (SpeedMill PLUS, Analytik Jena AG) containing 1 mL RNA Soil Lysis Solution (provided by the kit). After two cycles of homogenization, each for 3 min, the solution was clarified by centrifugation at 12.000 g for 5 min. The supernatant was transferred to RNase-free Eppendorf tubes and vortexed with 750 μL of phenol: chloroform (1:1, v/v). After incubation for 5 min and centrifugation at 12.000 g for 5 min, 200 μL Inhibitor Removal Solution (provided by the kit) were added to the aqueous phase. After centrifugation at 14.000* g* for 5 min, the supernatant was treated with 660 μL isopropanol. The RNA was precipitated by centrifugation (14.000 g, 4 °C, 15 min), washed with 500 μL of 75% ethanol, dried and dissolved in 200 μL RNase-free water. To this solution 600 μL of RNAMATRIX Binding Solution and 10 μL of RNAMATRIX slurry were added. The mixture was vortexed briefly and incubated for 5 min in a rotary overhead shaker with gentle vertical rotation, which allowed binding of RNA to the matrix. After centrifugation for 10 s, the pellet was washed twice with 500 μL RNAMATRIX Wash Solution. The supernatant was completely removed, the pellet air dried 5 min at room temperature and resuspended in 50 μL RNase-free water. Two to seven µg of total soil RNA were isolated from 0.5 g soil. Quality and yield of the isolated total RNA preparations were determined based on the extinction values at 260 and 280 nm. Only samples with an E260 / E280 ratio of more than 1.6 and lower than 2.0 were used for synthesis of cDNAs.

### cDNA synthesis and quantitative real-time PCR for the detection of *P. betae*, sugar beet viruses and SBWMV

Reverse transcription was performed using M-MLV Reverse Transriptase (Promega) according to the manufacturer’s instructions. To approximately 300 ng of total RNA, 29 μL enzyme-free reverse transcriptase premix (RT-Premix) were added. Premix contained the manufacturer-supplied buffer (M-MLV Reverse Transcriptase, Promega), 0.5 mM dNTPs and the sequence-specific primer (0.4 μM). cDNAs were synthesized with specific primers (Table S1). The reaction mixture was incubated at 72 °C for 2 min, cooled to room temperature, mixed with 10 U RNase inhibitor and 100 U M-MLV Reverse Transcriptase and incubation at 42 °C for 60 min.

Quantitative real-time PCR (qPCR) was conducted to detect *P. betae* and viral cDNAs. Each 20 µL reaction included 2 µL of cDNA, 1 × BioCat SensiFast SYBR No-Rox Master Mix (2x) and 150 nM of specific forward and reverse primer. The tubes were incubated for 5 min at 95 °C, followed by 35 cycles (10 s at 95 °C, 20 s at 65 °C, 30 s at 72 °C). Specific melting temperature was determined for each amplified virus-specific amplicon during real-time PCR in a qTower2.2 thermocycler (Analytik Jena AG).

### cDNA synthesis and quantitative real-time PCR for the detection of sugar beet housekeeping genes

For the detection of housekeeping genes in sugar beet roots an additional DNase treatment was performed after RNA-extraction. The TURBO DNA-free™ Kit (Thermo Fisher Scientific, Waltham, MA, USA) was used according to the manufacturer’s instructions. 2.200 ng RNA was mixed with Turbo DNase™ buffer (1x), the TURBO DNase™ enzyme (0.8 U/4.000 ng RNA) and sterile Milli-Q-H_2_O to 20 μL. After incubation for 30 min at 37 °C, 0.2 µL DNase inactivation reagent were added. The inactivation was performed for 5 min at room temperature, followed by 2 min centrifugation at 10.000 g. From the supernatant 300 ng was used in cDNA synthesis. For cDNA synthesis the ProtoScript® II reverse Transcriptase kit (NEB, Frankfurt, Germany) was used according to the manufacturer’s instructions. 300 ng RNA was mixed with ProtoScript II buffer (1x), random hexamer primer (6 µM), DTT (0.01 M), dNTP mix (0.5 mM), ProtoScipt II Reverse Transcriptase enzyme (200 U/μL) in a 20 µL reaction volume. cDNA synthesis was performed 5 min at 25 °C followed by 60 min at 42 °C and an inactivation step for 20 min at 65 °C. One µL cDNA was then used in qPCR by using the LUNA®universal qPCR Master Mix kit (NEB) by adding LUNA® Universal qPCR mix (1x), 0.25 µM forward and reverse primer in a 20 µL reaction volume. The qTower2.2 thermocycler (Analytik Jena AG) was used by running following program, 1 min 95 °C, 40 cycles of 15 s at 95 °C and 30 s at 60 °C. The housekeeping genes sugar beet elongation factor 1 β (EEF1B2, NM_001303081.2) and glyceraldehyde-3-phosphate dehydrogenase (GAPDH, XM_010679634.2) (Wetzel et al. 2021) (Table S1) were analyzed.

### Statistical analysis of the results

Student’s t-test was performed to determine the statistical significance of the results. For the determination of virus and *Polymyxa* quantities in roots, at least three biological replicates were analyzed per condition and time point. Quantification of *P. graminis* RNA in soil samples was made in two biological replicates. Photographs are representative images of independent observations.

## Results

### Seed-treatment with GE or Rha protects sugar beet against virus infection

**Fig. 1:**
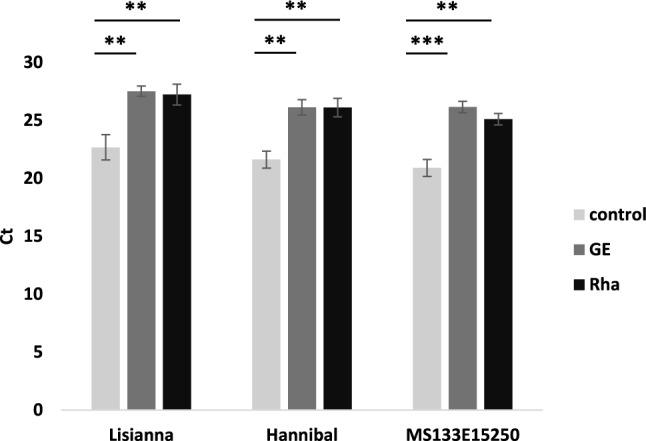
Antiviral protection by GE and Rha in different sugar beet varieties. BNYVV RNA4 was quantified by RT-qPCR in sugar beet roots of the varieties Hannibal, Lisianna and MS 133E15250, 5 wps of GE-, Rha- or control-treated seeds into soil from Pithiviers. Seeds were incubated for 8 h with 1:10 diluted GE-solution, 40 g L^−1^ Rha or water as control. Bars represent mean values ± SD, *n* = 3. Asterisks mark significant differences according to Student’s t-test: **, *P* < 0.01; ***, *P* < 0.001

To investigate whether seed-treatment with GE or Rha was effective to protect sugar beet plants against infection with BNYVV, seeds were treated for 8 h with 1:10 diluted GE-solution, 40 g L^−1^ Rha or water as control, planted into virus-containing soil from Pithiviers (France) and cultivated in the climate chamber. Five weeks post sowing (wps) lateral roots were sampled from seedlings which had emerged from the seeds and virus present in the roots was quantified by RT-qPCR. Seed treatment with GE and Rha resulted in a significant protection against BNYVV (Fig. 1). Ct values for the specific detection of BNYVV RNA4 increased between 4.5 and 5.3 Ct values in roots of GE-treated compared to water-treated samples and between 4.2 and 4.6 Ct values in Rha-treated vs. water-treated samples. Antiviral protection was independent of the sugar beet cultivar used as the effect of treatment with GE or Rha was similarly effective for each cultivar tested (Fig. 1).

**Fig. 2:**
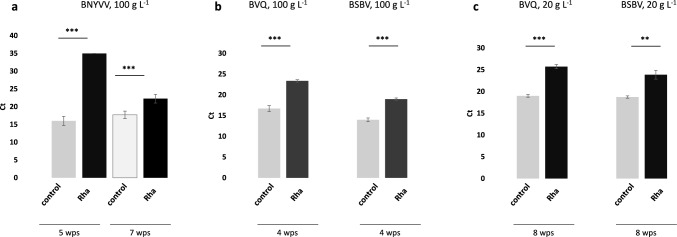
Antiviral protection by Rha at different time points after sowing and against different viruses. BNYVV RNA4, BVQ RNA2 and BSBV RNA3 was quantified by RT-qPCR in sugar beet roots grown in soil from Mintraching for the time period indicated below the graphs. Pelleted Seeds were incubated for 16 h with 100 g L^−1^ Rha, 20 g L^−1^ Rha, or water as control. Bars represent mean values ± SD. Asterisks mark significant differences according to Student’s t-test: **, *P* < 0.01; ***, *P* < 0.001. For samples in which no Ct values were obtained after 35 cycles by RT-qPCR a value of 35 was set for the calculation of Student’s t-tests. **a** and **b** Three biological replicates were analyzed per condition. **c** Replicates were from different RNA and cDNA preparations from a bulked sample in which a total of 24 seedlings grown in eight independent pots was combined

For treatment with Rha, we tested whether the antiviral effect changed over time. Pelleted sugar beet seeds were incubated for 16 h with 100 g L^−1^ Rha or water as control, planted into virus-containing soil from Mintraching (Germany) and cultivated in the climate chamber. RT-qPCR analysis after 5 and 7 wps revealed significant antiviral protection by Rha at both time points (Fig. 2a). However, the antiviral protection effect was stronger at the earlier time point. At five wps no BNYVV RNA was detected in roots of plants grown from treated seeds by RT-qPCR, while the control-treated plants contained the virus. At seven wps, Rha-treated plants contained the virus, however, the virus concentration in roots of plants which had received treatment was still 4.5 cycles lower compared to the control. The weaker protection effect at the later time point may suggest that virus titer can catch up with time as initial infection is not abrogated by the seed treatment. To which extent this would influence symptoms and yield losses needs to be investigated.

Rha-treatment of sugar beet seeds for 16 h at the concentration of 100 g L^−1^ also protected efficiently against infection by BVQ and BSBV. Ct values obtained for BVQ RNA2 and BSBV RNA3 from roots of Rha-treated sugar beet seedlings grown in virus-containing soil for four weeks were 6.6 (BVQ) and and 5.0 Ct-values (BSBV) higher compared to those obtained for RNA from roots of control plants (Fig. 2b). Also lower concentrations of Rha were effective in antiviral protection, 16 h incubation of pelleted seeds in 20 g L^−1^ Rha was still effective in protecting plants grown from these seeds against BVQ and BSBV 8 wps (Fig. 2c). However, no protective effect was observed with this concentration against BNYVV 7 wps (Fig. S1).

To verify that the quality of RNAs obtained from root tissue was not influenced by the seed treatment with GE and Rha, respectively, we analyzed the expression of the housekeeping genes GAPDH and EF2E1 in roots of plants grown from seeds in Mintraching soil for seven weeks after treatment. No significant difference in the expression of these genes was observed in roots of plants, where seeds had received treatment for 16 h with GE diluted 1:10 in water and 20 g L^−1^ Rha, respectively, compared to the water-treated control (Table S2).

### Seed treatment with GE or Rha does not reduce the amount of *P. betae* in sugar beet roots

**Fig. 3:**
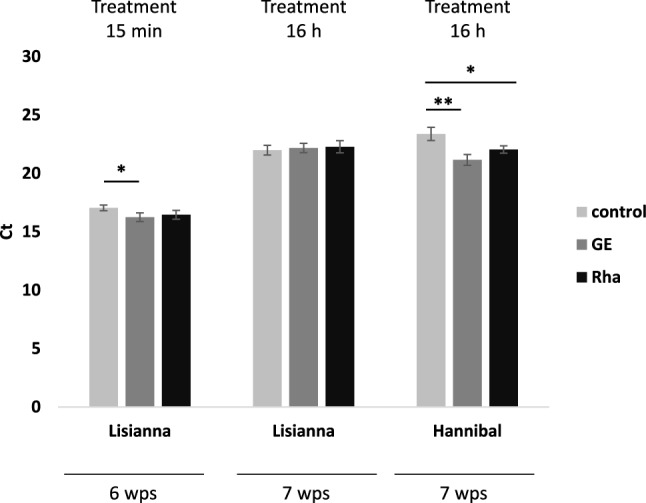
Quantification of *P. betae* RNA in sugar beet roots after seed-treatment with GE or Rha. RT-qPCR was conducted at 6 or 7 wps, respectively. Seeds were incubated in undiluted GE-solution, a solution of 200 g L^−1^ Rha or water as control for the indicated times and shown into soil from Pithiviers. Bars represent mean values ± SD, *n* = 3. Asterisks mark significant differences according to Student’s t-test: *, *P* < 0.05; **, *P* < 0.01

Seed treatment with the bio-substances GE and Rha may be directly inhibiting virus infection, but it may also be a consequence of an effect on the vector, *P. betae*. To investigate the latter possibility we quantified *P. betae* levels in roots of seedlings which emerged from seeds treated with GE, Rha or water and were grown in soil containing viruliferous *P. betae*. Interestingly, *P. betae* levels were slightly increased or remained unchanged six or seven wps into infected soil after incubation of the seeds with GE or Rha (Fig. 3). However, it cannot be excluded that seed treatment had inhibitory effects on early *P. betae* infection (several days after germination), which may have caused the observed antiviral activity of the bio-substances.

### Effect of GE and Rha on sugar beet growth and seed germination

**Fig. 4:**
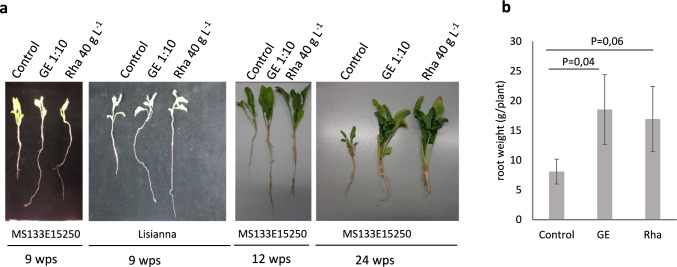
Influence of seed treatment with GE and Rha on sugar beet seeding development. **a** Phenotype of sugar beet seedlings grown in soil from Pithiviers (France). **b** Root weight of sugar beet seedlings at 6 wps into soil from Pithiviers. Bars represent mean values ± SD, *n* = 3. *P*-values according to Student ‘s t-test are depicted. Seeds had received treatment for 8 h with 1:10 diluted GE and 40 g L^−1^ Rha, respectively. Control seeds were treated with water

We analyzed seedling phenotypes after seed treatment with GE and Rha. Treatment with the bio-substances did not have a negative effect on sugar beet seedling phenotypes with the tested concentrations of the bio-substances. By contrast, we rather observed better seedling growth including better-developed and longer roots in virus-containing field soil (Fig. 4).

To investigate to which end the seed treatment had an effect on germination, we calculated germination rates. We incubated sugar beet seeds with different concentrations of GE and Rha for different periods of time and scored the number of seedlings which emerged from pots after planting the seeds into field soil and cultivation in the climate chamber. Incubation with 1:10 diluted GE for 8 h resulted in 100% germination of pelleted sugar beet, while incubation for 16 h resulted in approximately 35% inhibition of germination (Table 1). For Rha, 100% germination was obtained after 8 h incubation in 20 g L^−1^ (Table 1). Concentrations of 40 g L^−1^ resulted in 25–35% inhibition of germination. Pelleted seeds supported higher concentrations of the bio-substances compared to non-pelleted seeds (Table S3). Especially for GE, germination was strongly impaired after 8 h and abolished after 16 h incubation in 1:10 diluted GE. Germination after incubation of unpelleted seeds in 40 g L^−1^ Rha was also reduced compared to pelleted seeds. Taken together, germination strongly depended on the concentration of GE and on the incubation times; higher concentrations or longer incubation times of GE inhibited germination. Inhibition of germination likely results from GE being an ethanolic extract. For incubation with Rha, germination depended mainly on the concentration (Tables 1, S3). This is presumably due to direct cytotoxic effects of highly concentrated RLs (Thakur et al. 2021; Platel et al. 2022).

**Table 1:** Comparison of germination rates upon treatment of pelleted sugar beet seeds with different concentrations of GE and Rha

Plant species	Substance	Incubation time	Concentration/dilution	Germination rate in % (number of treated seeds)
Sugar beet	GE	15 min	Undiluted	7% (60)
Sugar beet	GE	16 h	Undiluted	5% (60)
Sugar beet	GE	16 h	1:10	65% (28)
Sugar beet	GE	8 h	Undiluted	5% (60)
Sugar beet	GE	8 h	1:5	66% (30)
Sugar beet	GE	8 h	1:10	100% (28)^a^
Sugar beet	Rha	15 min	200 g L^−1^	37% (100)
Sugar beet	Rha	16 h	200 g L^−1^	15% (100)
Sugar beet	Rha	16 h	100 g L^−1^	70% (100)
Sugar beet	Rha	8 h	40 g L^−1^	65% (27)^a^
Sugar beet	Rha	8 h	20 g L^−1^	100% (30)^a^
Sugar beet	Rha	8 h	2 g L^−1^	100% (30)^a^

Percent germination rate is calculated relative to the water control for each condition. Sugar beet seeds of the genotype Lisianna were tested

^a^In addition to sugar beet seeds of the genotype Lisianna, also sugar beet seeds of the genotype Hannibal were tested and similar germination rates were obtained

### Seed treatment with GE and Rha protects winter wheat from virus infection

**Fig. 5:**
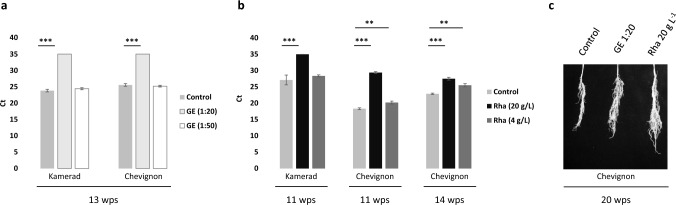
Antiviral protection by GE and Rha in winter wheat. SBWMV RNA2 was quantified by RT-qPCR in roots of different winter wheat cultivars grown in SBWMV-containing soil from Elxleben (Germany) for the indicated times. Seeds were incubated for 9 h with GE diluted 1:20 and 1:50 in water, respectively (**a**), or with Rha at a concentration of 20 g L^−1^ or 4 g L^−1^ (**b**). Control seeds were incubated for 9 h in water. *P*-values relative to control (Student’s t-test) are indicated above the bars (mean values ± SD, *n* = 3). **, *P* < 0.01; ***, *P* < 0.001. **c** Root phenotype of winter wheat cv. Chevignon seedlings 20 wps into soil from Elxleben (Germany). Seeds had received treatment for 9 h with 1:20 diluted GE, 20 g L^−1^ Rha, or water

We also investigated whether seed treatment with GE and Rha protected winter wheat from infection with *P. graminis*-transmitted viruses. We tested different winter wheat varieties with different degrees of resistance against SBWMV. The winter wheat cv. Kamerad contains no known resistance against SBWMV, while cv. Chevignon contains the *Sbm2* gene. Seed-treatment with GE and Rha led to reduced levels of virus accumulation in wheat plants which were grown in virus-containing soil. GE showed significant antiviral effects when seeds were incubated for 9 h in a GE solution diluted 1:20 (Fig. 5a). However, when GE was applied at a dilution of 1:50, no significant antiviral effect was observed anymore at 13 wps. Seed incubation for 9 h in 20 g L^−1^ Rha also resulted in significant antiviral effects; the plants grown from the seeds contained much less virus 11 wps and 14 wps (Fig. 5b). After treatment of seeds with a concentration of 4 g L^−1^ Rha, the protection effect against the virus was much reduced. Plant growth was not negatively affected by the treatment. By contrast, root length and root mass appeared to be increased in plants, which had received treatment with the bio-substances and were grown in virus-containing soil (Fig. 5c). Analysis of germination in winter wheat confirmed the observations from sugar beet that germination depended on the concentration of the biosubstances (Table 2). 25% to 50% reduced germination rates were obtained for winter wheat seeds treated with 1:20 diluted GE solution and 100 g L^−1^ Rha for 9 h. The lower tested concentrations did not negatively affect seed germination.

**Table 2:** Comparison of germination rates upon treatment of winter wheat seeds with different concentrations of GE and Rha

Plant species	Substance	Incubation time	Concentration/dilution	Germination rate in % (number of treated seeds)
Winter wheat	GE	9 h	1:10	0% (21)
Winter wheat	GE	9 h	1:20	50% (21)–75% (21)
Winter wheat	GE	9 h	1:100	100% (21)
Winter wheat	Rha	9 h	100 g L^−1^	50% (21)
Winter wheat	Rha	9 h	20 g L^−1^	100% (21)
Winter wheat	Rha	9 h	2 g L^−1^	100% (28)

Percent germination rate is calculated relative to the water control for each condition. Seeds of the genotypes Alcedo and Reform were tested and comparable germination rates were obtained

### GE and Rha affect *P. graminis* accumulation in soil and in roots when directly applied to the soil

To investigate if the bio-substances could effectively work against *Polymyxa graminis*, we irrigated barley host plants grown in soil containing viruliferous *P. graminis* with specific concentrations of GE and Rha, respectively, for 14 weeks. Subsequently*, P. graminis* was quantified in the barley roots and in the soil samples which had received the treatment.

**Fig. 6:**
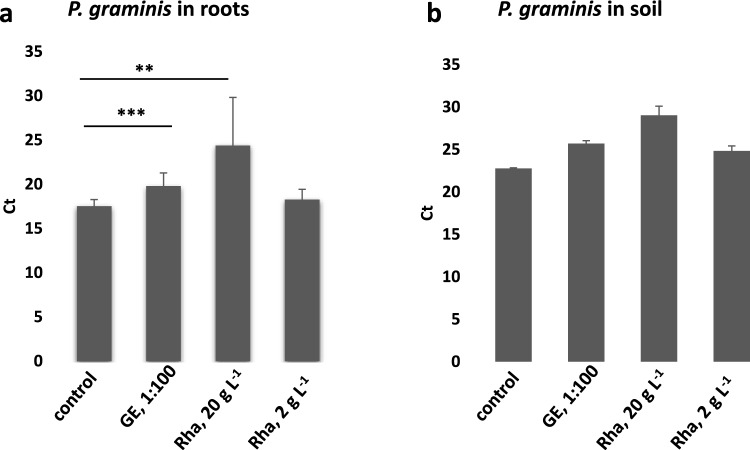
*P. graminis* accumulation in roots and soil upon irrigation with GE and Rha. **a** *P. graminis* DNA was quantified by qPCR in roots of barley plants grown in soil, which was irrigated with GE or Rha-containing solutions or water as control for 14 weeks. **b** *P. graminis* RNA was quantified by RT-qPCR directly in the irrigated soil. Irrigation was once a week with a fixed quantity of water, GE diluted 1:100 in water, or water containing a final concentration of 20 g L^−1^, or 2 g L^−1^ Rha, respectively. **a** Mean values ± SD, *n* ≥ 6. Asterisks mark significant differences according to Student’s t-test. **, *P* < 0.01; ***, *P* < 0.001. **b** Mean values ± SD,* n* = 2

The amount of *P. graminis* detected in the roots of plants grown in the soil which had received treatment with 1:100 diluted GE or 20 g L^−1^ Rha was significantly lower compared to the control (Fig. 6a). Consistently, treatment with 1:100 diluted GE resulted in an increase of the Ct values for *P. graminis* of 2,9 cycles, and treatment with 20 g L^−1^ and 2 g L^−1^ Rha resulted in an increase of the Ct values for *P. graminis* of 6,7 and 2,0 Ct values, respectively, in the soil (Fig. 6b). Thus, the bio-substances efficiently reduce the amount of *P. graminis* in roots and in soil, when directly applied to the soil. Whether this effect was due to a direct cytotoxic effect of the substances on the *P. graminis* zoospores or due to the induction of immunity in the host plants needs to be further addressed.

## Discussion

Plant protection or biocontrol agents work through direct cytotoxicity towards the target organism, through stimulation of immunity or promotion of plant growth or through a combination of these mechanisms. A whole set of such substances is currently assayed for their potential to promote plant growth and protect against biotic and abiotic stress (Galli et al. 2024). GE and Rha are among these plant protection agents. GE and RL have been shown to act through direct cytotoxicity towards oomycetes and fungal diseases and by stimulating plant immunity (Varnier et al. 2009; Sanchez et al. 2012; Botcazon et al. 2022; Hermann et al. 2022; Platel et al. 2022; Porsche et al. 2023). Whether these bio-substances can also effectively protect against virus infection in plants has not been investigated. Only one study analyzed the use of foliar applied RLs on virus infection (Haferburg et al. 1987). For tobacco mosaic virus, treatment of leaves with RLs reduced the number of local lesions produced on the local lesion host plant *Nicotiana glutinosa* up to 90%; and potato virus X infection and red clover mottle virus systemic infection of susceptible host plants was inhibited by foliar application of RLs to different extent (Haferburg et al. 1987). We here show that GE and Rha applied to seeds efficiently protect against infection with a number of *P. betae* and *P. graminis*-transmitted viruses in sugar beet and wheat. Both, GE and Rha, have been shown to induce plant immune responses (Sanchez et al. 2012; Hermann et al. 2022; Porsche et al. 2023) which may result in protection against virus infection. The antiviral protection observed after seed treatment of sugar beet and wheat seeds is independent of the variety and hence, independent of the presence of a resistance gene. This is specifically important in sugar beet, as resistance mediated by the widely employed *RZ1* gene is being broken (Liebe et al. 2023). Because of increased selection pressure by growing resistant cultivars, it is especially important that also resistant cultivars are efficiently protected against infection by plant protection agents. In wheat, the two known resistance genes *Sbm1* and *Sbm2* confer a translocation resistance, i.e. reducing virus translocation from roots to shoots (Kanyuka et al. 2004a; Bruschi et al. 2023). However, the roots of resistant plants still can become infected by the virus and vector, so that the introduction of virus and viruliferous *P. graminis* can still take place when resistant varieties are being cultivated. Hence, also for wheat production virus control strategies apart from resistant plants are urgently needed.

Despite germination was negatively affected to a certain extent at specific incubation conditions, seed treatment with GE and Rha promoted plant growth in virus-containing soil, either as a consequence of the plant protection properties or as a consequence of the intrinsic biochemical features of the components. A positive effect of bio-surfactants on plant growth after seed treatment has been observed (Adnan et al. 2018; Marchut-Mikołajczyk et al. 2021; Silva et al. 2024) and has been related to increasing the availability of nutrients and the amount of chlorophyll and polyphenols.

As GE and RLs are known to protect plants against fungi and oomycete pathogens, it is surprising that the substances do not show protection against *P. betae* after seed treatment. However, the missing plant protection effect against *P. betae* by seed treatment may be due to the fact that infection of roots is a continuous process (Decroës et al. 2022), so that an initial reduction of *P. betae* infection of roots at early stages after germination may have been compensated at the time of analysis, six to seven wps. That GE and Rha can act against *Polymyxa sp*. is demonstrated by our experiments, where we applied the bio-substances directly to soil.

In conclusion, seed treatment with GE and Rha is a cost efficient and easy applicable method to achieve significant protection against infection by soil-borne viruses in sugar beet and wheat. The bio-substances can be applied directly before sowing to commercially available seeds in a non-toxic procedure. Seed treatment can be applied to pelleted seeds. The bio-substances likely can be incorporated into the pelleting procedure and are compatible with seed dressing, as RLs have already been used in seed coats (Da Silva et al. 2015; Sancheti and Ju 2019). Moreover, application of GE and Rha to contaminated soil may be an option to combat infection with *Polymyxa* and other plasmodiophorid parasites. Future studies will investigate whether seed treatment with the biosubstances also effectively protects plants from soil-borne virus infection in the field. Moreover, future studies will focus on the mechanism, by which these substances induce plant protection and investigate, if seed treatment with the bio-substances also protects against infection with insect-transmitted plant viruses.

## Supplementary Information

Below is the link to the electronic supplementary material.

Supplementary file1 (DOCX 35 KB)

## Data Availability

All data supporting the findings of this study are available within the paper and its Supplementary Information.

## Abbreviations

BNYVV: Beet necrotic yellow vein virus
BSBV: Beet soil borne virus
BVQ: Beet virus Q
GE: *Glycyrrhiza glabra* extract
*P. betae*: *Polymyxa betae*
*P. graminis*: *Polymyxa graminis*
Rha: Rhapynal
RL: Rhamnolipid
SBWMV: Soil-borne wheat mosaic virus
wps: Weeks post sowing
