# Supplementary material for: Bio-control of soil-borne virus infection by seed application of Glycyrrhiza glabra extract and the rhamnolipid Rhapynal
Source: Planta. 2024 Sep 13;260(4):94. doi: 10.1007/s00425-024-04529-5 (PMC11399307; doi:10.1007/s00425-024-04529-5)
Supplement: Supplementary file 1 — Supplementary file1 (DOCX 35 KB) [file 425_2024_4529_MOESM1_ESM.docx]

**Supplemental Material**

**Bio-control of soil-borne virus infection by seed application of *Glycyrrhiza glabra* extract and the rhamnolipid Rhapynal**

Viktoria Fomitcheva, Claudia J. Strauch, Sabine Bonse, Petra Bauer, Thomas Kühne, and Annette Niehl

Julius Kühn Institute (JKI) – Federal Research Centre for Cultivated Plants, Institute for Epidemiology and Pathogen Diagnostics, Messeweg 11-12, Braunschweig, Germany

Corresponding author: [Annette.niehl@julius-kuehn.de](mailto:Annette.niehl@julius-kuehn.de)

Table S1. Primers and probes used in quantitative real-time PCR

| Primer name | Sequence | Reference |
| --- | --- | --- |
| Primers for the quantification of *P. graminis* DNA in real-time PCR | | |
| PxRealF | CGTCGCTTCTACCGATTGGT | Ward et al. 2005 |
| PxRealR | CCTTGTTACGACTTCTTCTTCCTCTAGT |  |
| PxRealP | FAMCCGGTGAACAATCGMGB |  |
| Primers for the quantification of *P. betae* RNA in real-time two step RT-PCR* | | |
| Pb-for-5' | ACGTTACTGGAGACCAAC |  |
| Pb-rev1-3' | CTGTCATCATATCCTTTAGAC |  |
| Primers for the quantification of BNYVV RNA in real-time two-step RT-PCR* | | |
| RNA1-Fw | TTGCGGAATTGTTGAGTGCG |  |
| RNA1-Rv | TCCCTGATAGGGTCAGCCAA |  |
| RNA2-Fw | ACAATCGCATGCTATGGACT |  |
| RNA2-Rv | AGCAGCAGCTAATTGCTAT |  |
| RNA3-Fw | CACGGTGTTCGGTGAGAAGA |  |
| RNA3-Rv | CGGACGGGAACACCATGTAA |  |
| RNA4-Fw | TGCCCCGTGTTATAGTGGTG |  |
| RNA4-Rv | CAAAACCTTCGCCACCCTTC |  |
| RNA5-Fw | GCCGCTTTTCCTTTGCGATT |  |
| RNA5-Rv | CGGGGGACCAACAAACATCA |  |
| Primers for the quantification of BVQ RNA in real-time two-step RT-PCR* | | |
| BVQ-Fw | GCGCATTGGACCGCGGAACAT |  |
| BVQ-Rv | TGAGCCGGTCCACTTCAATCC |  |
| Primers for the quantification of BSBV RNA in real-time two-step RT-PCR* | | |
| BSBV-Fw | ATGGATCCTCCAGCAATAATA |  |
| BSBV-Rv | TTAGAAACGACACTTTACCAC |  |
| Primers for the quantification of SBWMV RNA in real-time two-step RT-PCR* | | |
| PGRV4a | CTGCGACTCACGCTTACATA |  |
| PGRV4b | TAACCGCTTTGGGATGATAG |  |
| Primers for the quantification of *Beta vulgaris* GAPD and EF2 E1 RNA in real-time two-step RT-PCR^§^ | | |
| BvEF2_E_1 For | AGCTGCGAAAATGGTGAAGT | Wetzel et al. 2021 |
| BvEF2_E_1 Rev | AGCGTTGATTTCCCGTGATC |  |
| BvGAPDH For | CACCACCGATTACATGACATACA |  |
| BvGAPDH Rev | GGATCTCCTCTGGGTTCCTG |  |

*, reverse primers were used for cDNA synthesis; ^§^, random oligomers were used for cDNA synthesis.

WARD, E., KANYUKA, K., MOTTERAM, J., KORNYUKHIN, D. & ADAMS, M. J. 2005. The use of conventional and quantitative real-time PCR assays for *Polymyxa graminis* to examine host plant resistance, inoculum levels and intraspecific variation. *New Phytologist,* 165**,** 875-885. <https://doi.org/10.1111/j.1469-8137.2004.01291.x>

WETZEL, V., WILLLEMS, G., DARRACQ, A., GALEIN, Y., LIEBE, S. & VARRELMANN, M. 2021. The *Beta vulgaris*-derived resistance gene Rz2 confers broad-spectrum resistance against soilborne sugar beet-infecting viruses from different families by recognizing triple gene block protein 1. *Molecular Plant Pathology,* 22**,** 829-842. <https://doi.org/10.1111/mpp.13066>

Table S2. Housekeeping gene expression in sugar beet roots upon seed treatment with GE or Rha.

| Treatment  of seeds | Concentration/dilution; incubation time | Time point (wps) | cultivar | soil | Plant gene detected | Ct-value treatment  (mean value ± SD, *n* =3) | Ct-Value  control  (H_2_O) (mean value ± SD, *n* =3) | *P*-value t-test |
| --- | --- | --- | --- | --- | --- | --- | --- | --- |
| GE | 1:10; 16 h | 7 | MS | M | *B. vulgaris* GAPDH  *B. vulgaris*  EEF1B2 | 29.39 ±  0.29  31.65 ±  0.28 | 29.64 ±  0.05  31.73 ±  0.54 | 0.38  0.57 |
| Rha | 20 g L^-1^; 16 h | 7 | MS | M | *B. vulgaris*  GAPDH  *B. vulgaris*  EEF1B2 | 29.55 ± 0.32  31.28 ±  0.34 | 29.64 ±  0.05  31.73 ±  0.54 | 0.66  0.35 |

GE, *Glycyrrhiza glabra* extract; Rha, rhapynal; MS, cv. MS133E15250; M, Mintraching; GAPDH, glyceraldehyde-3-phosphate dehydrogenase; EEF1B2, elongation factor 1 β.

Table S3. Comparison of germination rates upon treatment of non-pelleted sugar beet seeds with GE and Rha. Percent germination rate is calculated relative to the water control for each condition. Seeds of the cultivars Lisianna (Lis), Hannibal (Han) and MS133E15250 (MS) were tested.

| Plant species | Substance | Incubation time | Concentration/ dilution | Germination rate in %  (number of treated seeds) |
| --- | --- | --- | --- | --- |
| Sugar beet cv. Lis | GE | 16 h | 1:10 | 0% (28) |
| Sugar beet cv. Han/MS | GE | 8 h | 1:10 | 7% (28) |
| Sugar beet cv. Lis | Rha | 16 h | 40 g L^-1^ | 47% (30) |
| Sugar beet cv. Han/MS | Rha | 8 h | 40 g L^-1^ | 46% (30) - 55% (30) |


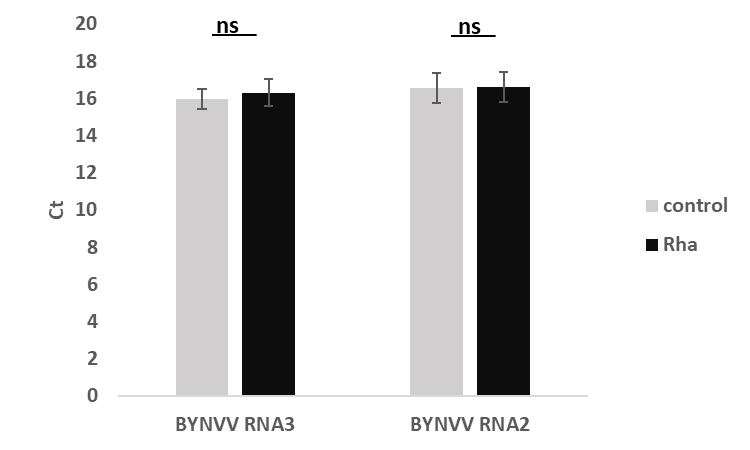


**Fig.** S1. BNYVV accumulation after seed treatment with 20 g L^-1^ Rha. BNYVV RNA2 and RNA3 were quantified by RT-qPCR in sugar beet roots 7 wps of Rha- or control-treated seeds into soil from Pithiviers. Pelleted Seeds were incubated for 16h with 20 g L^-1^ Rha or water as control. Bars represent mean values ± SD, *n* = 3. ns, non significant.
